# Barbed suture in neck dissection: a randomized clinical study on efficacy, safety and aesthetic outcome

**DOI:** 10.1007/s00405-024-08869-6

**Published:** 2024-08-02

**Authors:** Johannes Doescher, Benjamin Emmanuel, Jens Greve, Patrick J. Schuler, Fabian Sommer, Simon Laban, Johannes Veit, Thomas K. Hoffmann

**Affiliations:** 1 Department of Otorhinolaryngology, Head and Neck Surgery, University Medical Center Ulm, Ulm, Germany; 2 Department of Otorhinolaryngology, University Hospital Augsburg, Sauerbruchstraße 6, 86153 Augsburg, Germany; 3 Nasenchirurgie München, Munich, Germany

**Keywords:** Barbed suture, Neck dissection, Cost analysis, Surgical training

## Abstract

**Purpose:**

The resection of lymph nodes/neck dissection is a typical part of the surgical treatment of head and neck malignancies. The aim of this study was to compare subcutaneous closure using single knotted, braided suture (Vicryl^TM^, standard arm) with continuous self-locking, monofilament barbed suture (V-Loc^TM^, experimental arm).

**Methods:**

Neck Lock was a randomized clinical trial at a single tertiary referral center. It was conducted from 2016 till 2022 with a follow-up period of 3 months. Assessment of safety and aesthetic outcome was double-blinded. 68 patients were randomized after application of exclusion criteria. Subcutaneous wound closure was performed in an intrapatient randomized fashion for suture technique. The primary endpoint was the duration of subcutaneous sutures. Wound healing and scar formation were recorded at multiple postoperative intervals as secondary endpoints.

**Results:**

The median age was 61 years, 89.7% were male. 92.6% suffered from a squamous cell carcinoma. There was a significant difference in median subcutaneous suture time (p = 0.024) between the experimental (6:11 ± 2:30 min) and standard (7:01 ± 2.42 min) arms. There was no significant difference in safety when assessing adverse events (AEs). At least one AE occurred in 14.7% vs. 5.9%, for barbed and smooth sutures respectively (p = 0.16).

**Conclusion:**

For neck dissection of head and neck malignancies, subcutaneous wound closure with self-locking sutures offers significant time savings over the single knot technique with similar safety and aesthetic results.

**Trial registration information:**

The trial was registered with WHO acknowledged primary registry “German Clinical Trials Register” under the ID DRKS00025831 (https://drks.de/search/de/trial/DRKS00025831).

**Supplementary Information:**

The online version contains supplementary material available at 10.1007/s00405-024-08869-6.

## Introduction

Neck dissection, a surgical procedure commonly used in the management of various head and neck pathologies [1], presents unique challenges for wound closure due to the complex anatomy and functional importance of the neck region. Traditionally at our center, this is achieved with a subcutaneous self-dissolving braided suture (e.g. Vicryl™, Polyglactin suture) using a single knot technique. This requires about 15 single sutures of 3 knots each, which may take a considerable amount of time. Individual knots may also come loose, causing dehiscence. Primary skin closure is then usually performed with staples or non-absorbable sutures.

Suturing techniques have evolved over the years, with conventional sutures remaining the standard for wound closure. However, with numerous potential advantages over conventional sutures, the introduction of barbed sutures has revolutionized the field of surgical wound closure [2]. Barbed sutures, also known as self-retaining sutures or knotless sutures, are an innovative type of surgical suture that are equipped with unidirectional barbs that provide anchoring and tension distribution capabilities. Unlike conventional sutures, which require meticulous knot tying to maintain tension and secure wound edge approximation, barbed sutures are designed to engage and lock into tissue, holding it in place without the need for knots [3].

In neck dissection, meticulous wound closure is of paramount importance as it has a direct impact on postoperative complications such as wound dehiscence, hematoma formation and infection [4]. These complications can significantly increase length of hospitalization, delay adjuvant treatments, and negatively impact overall patient outcomes. In addition, the unique anatomy of the neck presents challenges in achieving uniform and stable wound closure, further emphasizing the need for innovative techniques [5].

Previous studies have highlighted the potential benefits of using barbed sutures in a variety of surgical procedures, including abdominal, gynecological and orthopedic surgery [6]. These knotless sutures allow for a certain amount of time saved. So far, in addition to experience in laparotomy, there are also observations in plastic surgery where the safety is equivalent to that of a conventional suture [7]. Even a reduction in wound seroma formation has been reported for latissimus dorsi flap donor sites [8]. In another study on abdominoplasty, suturing with barbed sutures resulted in significant time savings with the same level of safety [9].

In the market, Medtronic (Minneapolis, MN, USA) with its V-Loc™ system competes with Ethicon (Somerville, NJ, USA) with its STRATAFIX™ and Corza Medical (Westwood, MA, USA) with its Quill™ [2]. Until now, studies in the field of plastic skin closure have been performed only on the trunk of the body and, increasingly, in the field of minimally invasive facelift procedures [10]. However, there have been no studies to determine whether suturing neck dissection wounds with a barbed suture is more efficient and faster with the same level of safety as compared to a conventional subcutaneous suture.

The aim of this study was to compare the two methods of subcutaneous closure of neck dissection wounds in terms of time savings and aesthetic and functional outcome, i.e. the wound healing process, and, if the study objectives are met, to establish the continuous subcutaneous suture in clinical practice in the future. The time savings in turn has the advantage for patients and cost bearers of a shortening of the operation time with generally faster convalescence and shorter length of stay as well as a reduction of the costs for the surgical intervention [11, 12].

## Material and methods

### Trial design

The study was set up as a prospective, single center, randomized, controlled trial designed to evaluate subcutaneous closure time, adverse events, and aesthetic results of absorbable barbed sutures (V-Loc™ 90, Medtronic, Minneapolis, MN, USA) compared with conventional absorbable braided suture (Vicryl™, Ethicon Inc., Somerville, NJ, USA) for closure of neck dissection wounds in patients suffering from head and neck malignancies. The assessment of safety and aesthetics was double-blinded, as neither the patient nor the examining doctor knew which side of the neck had been sutured with the barbed suture.

Randomization was 1:1 using the respective other neck side as the comparator by the investigators. One side was closed with a barbed suture whereas the contralateral side was sewed with braided sutures. In this manner, the patients served as their own control. The selection of wound closure material for each side (right versus left) was randomized (through prepared and sealed envelopes) for barbed suture or braided suture.

Sample size was determined following a review of the duration for subcutaneous wound closure of pilot cases in our own institution using an alpha of 5% and a power of 80%. The case number software nQuery 7.0 yielded a case number of 27 patients. Since the estimate was made without documented preliminary data, a more cautious planning with a mean difference of 3 min and a standard deviation of the differences of 8 min was made. These assumptions resulted in a case number of 58 patients. In order to be able to include the respective suture length per side of the neck as a covariate in the evaluation of the primary outcome measure, a total number of 70 patients was finally considered adequate.

### Participants

The monocentric study was conducted in a tertiary referral hospital. Prior to enrollment, each patient signed an informed consent. The study procedures were approved by the Ethics Committee of Ulm University (#301/15) and performed in accordance with the ethical standards of the Helsinki declaration of 1975. This study was registered with the German Clinical Trial Registry (DRKS00025831).

Patients with a head and neck malignancy who were recommended for curative tumor resection and bilateral neck dissection by multidisciplinary tumor board decision were eligible for inclusion in the study. Inclusion criteria were further age ≥ 18 and signed informed consent. If patients had previous surgery or radiotherapy of the neck or if a skin resection due to metastatic infiltration was necessary, patients were excluded.

### Intervention and outcome measures

Subcutaneously, one side of each patient’s neck was closed after neck dissection with braided suture (Vicryl™, 3–0, FS-2) using a single knot technique and the other side was closed with a continuous barbed suture (V-Loc™ 90, 3–0, P-12). An instruction video can be found in the supplementary material (Vid. S1). The skin closure was performed with staple sutures.

The primary objective was duration of suture. Therefore, on the day of the surgery, the level of training of the suturing physician (trainee or senior physician), length of the incision in centimeters and duration of the subcutaneous suture in minutes and seconds were recorded separately for both sides. To assess the secondary objectives of aesthetic appearance and safety, postoperatively, on day 2, 5 and 10, the presence of dehiscence, drainage insufficiency, crust formation, step formation and fistulae was documented in five degrees of severity: none, slight, moderate, distinct, very distinct.

Follow-up took place three to six months after the intervention and it was recorded whether the wound healing process was normal or whether any adverse event (AE) had occurred. Assessment of the aesthetic outcome of the neck dissection scars by the patient and the investigator using a simple score was done. Lastly, the scars were photographed.

### Statistics

Statistical comparison of standard and barbed sutures was performed using Wilcoxon matched-pairs signed rank test. Subgroup analyses were done with a Mann–Whitney-U-Test. Correlation analysis was calculated with Spearman correlation as values were not normally distributed. The proportion of complication found on each side (barbed vs. conventional suture) were evaluated with Fisher’s exact test. For aesthetic parameters across time points and final aesthetic evaluation by an independent physician and the patient a Chi^2^-test was used. A p-value < 0.05 was considered significant.

## Results

### Participants

**Fig. 1:**
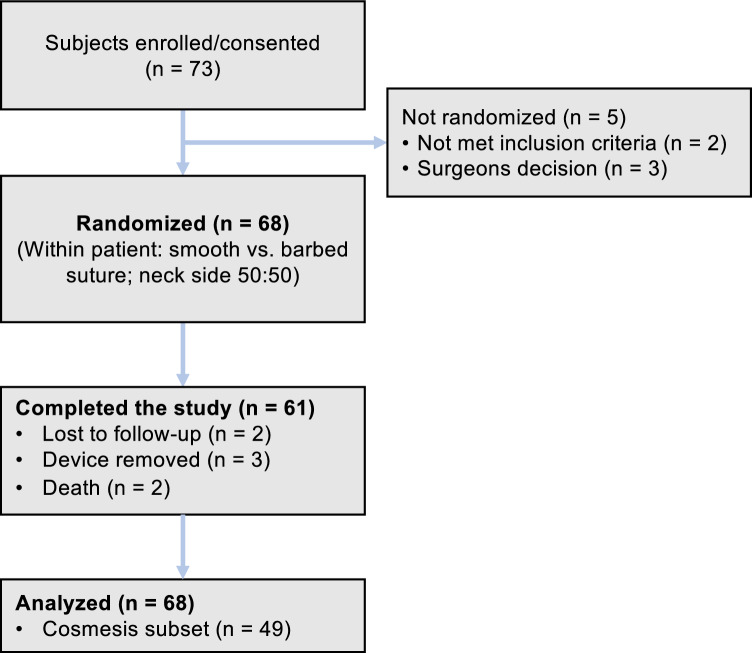
Consort diagram displaying patients’ flow in study

A total of 73 patients were consented and enrolled in the study from January 2016 until June 2022. Of those, 68 patients were randomized and treated. Five patients had to be excluded after enrollment due to missed in-/exclusion criteria or individual, intra-surgical decision, such as critical incidents which did not allow the surgical team to record study measures adequately and unplanned skin resection with the necessity of flap transplantation. All randomized patients were included in the efficacy and safety analyzes (n = 68). Only patients with complete data sets on aesthetic outcome were included in the cosmesis analysis (n = 49). Figure 1 provides a consort diagram of the study population.

### Clinical characteristics

A summary of clinical characteristics can be found in Table 1. As patients served as their own internal control, characteristics were identical for smooth and barbed sutures. Patients were of a median age of 61 years and predominantly male (89.7%). Most patients suffered from squamous cell carcinoma (92.6%), mainly in the oral cavity, pharynx and larynx. About half of the cases were N0. Risk factors for impaired healing were mainly smoking (80% of the patients), followed by high blood pressure (42.6%) and diabetes (14.7%). Only a minority of 4 patients were previously irradiated, all of them received neck dissection as part of a salvage laryngectomy.

**Table 1:** Clinical parameter of evaluable patients

Clinical parameter	Number of patients n = 68
Age (median)	61 ± 10 years
Gender	
Male	61 (89.7%)
Female	7 (10.3%)
Histology	
Squamous cell carcinoma	63 (92.6%)
Adenocarcinoma	3 (4.4%)
Adenoid cystic carcinoma	1 (1.5%)
Undifferentiated carcinoma	1 (1.5%)
Tumor site	
Oral cavity	11 (16.2%)
Oropharynx	18 (26.5%)
Hypopharynx	6 (8.8%)
Larynx	20 (29.4%)
Nasopharynx	1 (1.5%)
Nasal cavity and paranasal sinus	6 (8.8%)
Salivary gland	2 (2.9%)
Unknown primary	4 (5.9%)
T stage	
Tx	2 (2.9%)
T0	4 (5.9%)
T1	7 (10.3%)
T2	15 (22.1%)
T3	17 (25.0%)
T4a	23 (33.8%)
N stage	
N0	33 (48.5%)
N1	16 (23.5%)
N2a	5 (7.4%)
N2b	5 (7.4%)
N2c	3 (4.4%)
N3b	6 (8.8%)
M stage	
M0	68 (100%)
Side of barbed suture	
Right	34 (50%)
Left	34 (50%)
Concomitant diseases	
Arterial hypertension	29 (42.6%)
Diabetes mellitus	10 (14.7%)
Coronary heart disease	10 (14.7%)
Peripheral artery disease	5 (7.4%)
S/P apoplectic stroke	3 (4.4%)

### Wound closure time

**Fig. 2:**
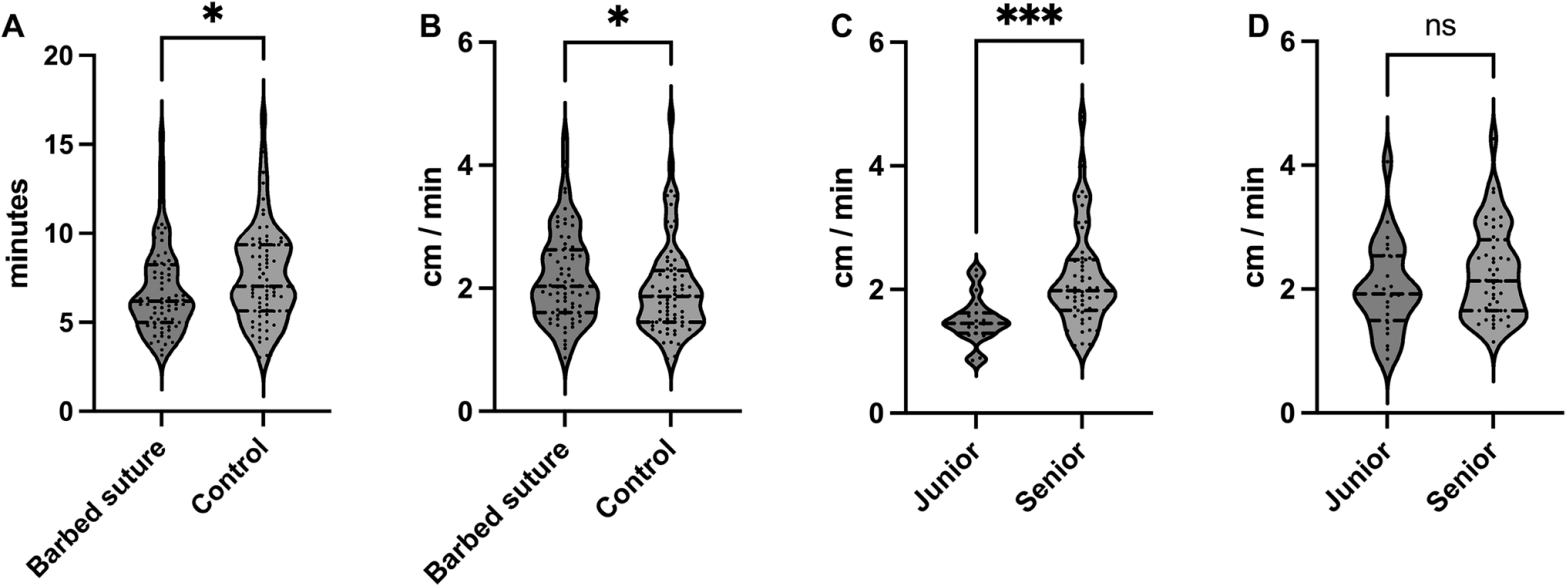
Violin plots showing **A** suture time for barbed and conventional sutures, **B** suture speed in cm/min, **C** suture speed for conventional sutures and **D** suture speed for barbed sutures stratified by training level

**Fig. 3:**
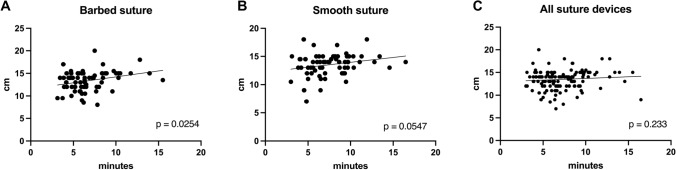
Scatter plots show correlation between incision length and suture time for **A** barbed sutures, **B** for smooth sutures and **C** for all devices together. Regression line indicates correlation trend

Median incision length, which needed to be closed was comparable for barbed vs. smooth side (14 cm, range 8–20 cm, vs. 14 cm, range 7–18 cm; p = 0.52). Subcutaneous wound closure was significantly faster with a median of 6:11 ± 2:30 min for barbed sutures as with interrupted sutures with 7:01 ± 2.42 min (p = 0.024, Fig. 2a). To avoid bias in length of incision, suturing speed was calculated in cm/min. First, correlation between incision length and wound closure time was analyzed. A significant correlation could be proven for wounds closed with barbed suture (r = 0.27, p = 0.025; Fig. 3a). A positive correlation, however not significant, between the two parameters could be calculated for smooth sutures (r = 0.23, p = 0.055; Fig. 3b). Median subcutaneous suturing speed stayed significantly faster in this analysis with 2.18 ± 0.74 cm/min for the barbed device compared to 1.99 ± 0.76 cm/min for the smooth device (p = 0.046, Fig. 2b). Another possible influence on suture speed is the training level of the individual surgeon. Barbed sutures were placed by junior doctors in 24 cases and by senior doctors in 44 cases. Conventional sutures were applied in 19 cases by junior doctors and in 49 cases by seniors. Even though Spearman analysis showed no significant correlation between incision length and suture time when stratifying for training level (Fig. S2), suture speed was analyzed to adjust for length of incision. When comparing subcutaneous suturing speed between consultants and trainees using conventional suture devices, there was a highly significant difference (median of 1.98 ± 0.79 cm/min vs. 1.45 ± 0.37 cm/min, p < 0.001, Fig. 2c). There was no difference regarding training level with barbed sutures (median of 2.14 ± 0.73 cm/min vs. 1.92 ± 0.73 cm/min, p = 0.22, Fig. 2d).

### Safety

Follow-up was three months. 10 patients experienced at least one AE on the barbed suture side of the neck (14.7%), while 5.9% experienced at least one AE on the smooth suture side of the neck (n = 4). This difference was not significant (p = 0.16). AEs were mainly minor and, in the majority, not directly related to the suture device. The most common and significant AE directly related to barbed sutures was intraoperative filament breakage in 6 cases compared to none for smooth sutures (p = 0.03). Table 2 summarizes AEs at the subject and device level. There was no significant correlation between a pre-existing medical condition and the adverse events.

**Table 2:** Prevalence of adverse events according to suture device. Percentages are based on the number of total patients. Patients with multiple occurrences of the same event were counted only once within a specific reported term

Reported term	Barbed side (n = 68)	Conventional side (n = 68)	p-value
At least 1 adverse event	10 (14.7%)	4 (5.9%)	0.16
Rupture of suture	6 (8.8%)	0	0.03
Suture incomplete*	2 (2.9%)	0	0.5
Fistula	0	3 (4.4%)	0.24
Bleeding	3 (4.4%)	0	0.24
Seroma	2 (2.9%)	0	0.5
Revision due to residual disease	1 (1.5%)	1 (1.5%)	> 0.99
Suture extrusion	1 (1.5%)	0	> 0.99
Insufficient drainage vacuum	1 (1.5%)	0	> 0.99
Wound infection	1 (1.5%)	0	> 0.99

*Continued with conventional interrupted suture

### Aesthetics

Initial wound healing was assessed on days 2, 5 and 10 for three categories: crust formation, step formation and general appearance. Within 49 evaluable patients, there were no differences between neck sides sutured with barbed or smooth device. After three months, aesthetic appearance was evaluated by both, the patient and an independent physician. Again, there were no statistic significant differences between suture devices.

## Discussion

The study evaluated the efficacy and safety of barbed sutures for subcutaneous wound closure after neck dissection. As closure with barbed sutures is a widely used technique in a verity of surgical procedures, the study was aimed to close a respective lack of evidence for neck dissection. The main finding was a significantly faster wound closure time when barbed sutures were used. On average, the investigational technique was about 50 s faster than the control technique. Multiple studies on the application of barbed sutures have been published, focusing on various aspects, but predominantly evaluating aesthetic appearance. Barbed sutures are regularly utilized in a variety of surgeries including obstetric and gynecological, gastrointestinal, and orthopedic procedures [2]. These sutures can also be used in plastic surgery to tighten facial tissue through a minimally invasive approach [13]. In many of these surgeries, significant suturing of muscle and subcutaneous tissue is necessary, and the application of barbed sutures has been shown to significantly decrease the time required for closure [9, 14–20]. Reductions of closure time ranging from 40 to 50% have been reported, resulting in an overall reduction in operation time of 25% to 30%, dependent on the amount of stitches needed [21]. However, a meta-analysis of 17 randomized controlled trials revealed significant heterogeneity between various types of surgery and time savings [6]. As closure of the wound after neck dissection constitutes only about 10–20% of the total operation time and does not require any muscle sutures, the time saved by utilizing a faster subcutaneous suture technique may not be as significant. Notably, most studies examining the use of barbed sutures have solely measured the time required for wound closure without taking the incision length into consideration. This may introduce some bias; therefore, our study was specifically designed to address this issue. When applying this adjustment, wound closure with barbed sutures was 9.5% faster than with conventional devices.

Another potential confounding factor, not previously reported in other studies, is the level of surgical training. Thus, the surgeons were divided into two groups: those in training, also referred to as residents, fellows, and attending surgeons with at least 10 years of surgical experience. Interestingly, there was no significant difference in the closure time using barbed sutures between the two groups. However, junior doctors required significantly more time to complete wound closure using smooth sutures. This suggests that barbed sutures do not require extensive training to achieve similar sewing speed. One alternate interpretation could be that both training level groups are new to the use of barbed sutures, but only residents are “relatively” new to the use of interrupted sutures. This could be a confounding factor since consultants only have a slightly slower suturing speed with smooth sutures compared to barbed sutures.

Examining safety measures, few clinical events directly related to wound closure technique were recorded. The only substantial disparity between suture devices was intraoperative filament breakage. This happened in six cases where barbed sutures were used and resulted in two issues. Firstly, opening a second, costly barbed suture is required to complete the suture. Secondly, if the barbed suture line is interrupted, the wound may not be as tightly closed. However, there was no record of wound dehiscence or drainage insufficiency occurring more frequently in cases where filament breakage was reported. An animal study investigating biomechanical properties found significantly more instances of suture breakage when using V-Loc 90 in comparison to Quill or smooth suture devices [3]. The results of the present study are supported by a large retrospective analysis of wound complications. The authors found no significant difference in overall complication rates when comparing barbed sutures with conventional techniques. Only dermal wound separation was more common with barbed sutures [22].

Following the adverse event analysis, aesthetic outcomes were also evaluated. Although there was one suture extrusion on the barbed suture-closed side of the neck, this was not significant between devices and had no further impact on scar appearance ratings at follow-up. This has also been found in numerous studies, some previously cited, and was summarized in a systematic review by Motosko and colleagues in 2018. However, a more standardized approach to scar assessment is recommended, and a corresponding guideline has been proposed by the authors [23]. Accordingly, a limitation of the present study is the method used to assess scarring, as only about half of the patients could be evaluated and no standardized scar assessment questionnaire was used. In contrast, two other publications reported improved aesthetic outcomes with the use of barbed sutures. However, these studies were performed on leg wounds and abdominoplasty [24, 25]. According to the authors, these sites are known to be prone to excessive scarring, and barbed sutures appear to be able to reduce this risk.

A remaining question is whether the different suture materials may have an effect on scarring after adjuvant radiotherapy. Because most studies in the past did not evaluate the use of barbed sutures in oncologic patients, this question has not yet been addressed. In the present study, there was no difference in scar appearance during long-term follow-up, which was scheduled after adjuvant treatment. In 2019, we reported on a case of suture granuloma formation after adjuvant radiotherapy. The granulomas developed around the suture knots with the conventional suture material used in this study [26]. Since there are no knots using barbed sutures, this risk may be reduced.

Another limitation of the present study might be the use of single knot sutures as control. Tying the knot seems to be the prolonging factor in this technique compared to continuous suturing with barbed devices. Therefore, a more comparable technique could have been the use of smooth sutures in continuous application. As at our institution interrupted sutures are surgical standard, comparison of barbed sutures with continuous smooth sutures could have reduced the confounding effect of surgical experience. Even if this technique is done by individual surgeons, barbed sutures have consistently been compared with single-button sutures, as the risk of dehiscence is relevant with continuous subcutaneous sutures [6, 27].

Finally, cost issues need to be addressed. According to an analysis of surgical costs conducted by a German consulting firm, one minute in the operating room costs between 40 and 50 Euros [12, 28]. This means that using barbed sutures to close wounds on both sides of the neck could save between 80 and 100 Euros. On the other hand, the cost of the barbed suture device is higher. Depending on the volume discount, it can cost up to 45 Euros. However, generally only one device is needed per side of the neck, whereas 2 to 3 packs of conventional subcutaneous sutures are commonly used (around 5 € each). This results in a cost of approximately 15 Euros per side of the neck, which then translates into total cost savings of 26 to 46 Euros for the entire procedure when using barbed sutures. While this may seem like a very small amount, it must be put into perspective. If a department performs 100 bilateral neck dissections per year, the use of barbed sutures results in a cost savings of 2600 to 4600 Euros per year, which could be available for other investments. In addition, the robustness of the time savings between senior and junior physicians results in reduced wound closure time when performed by a trainee. These considerations are supported by other studies and summarized in a review focusing on total knee arthroplasty. It is important to note that cost savings are heterogeneous across studies and are mainly calculated by incorporating material costs and suturing time. However, there is no evidence to date of the influence of the duration of hospitalization and the treatment of complications on indirect costs [29].

## Conclusion

Barbed suture devices are a safe and efficient method for subcutaneous wound closure after neck dissection. Especially residents can use barbed sutures quickly and safely without prior training. Thus, barbed sutures can be safely used for subcutaneous wound closure of neck dissection incisions, but their overall benefit over conventional methods remains questionable as time saving and therefore cost saving may be clinically insignificant.

## Supplementary Information

Below is the link to the electronic supplementary material.

Supplementary file1 (PDF 211 KB)

Supplementary file2 (MP4 611825 KB)

## Data Availability

Original data can be obtained upon reasonable request to the corresponding author.
