# Supplementary figures and images for: Barbed suture in neck dissection: a randomized clinical study on efficacy, safety and aesthetic outcome
Source: Eur Arch Otorhinolaryngol. 2024 Aug 2;281(12):6613–20. doi: 10.1007/s00405-024-08869-6 (PMC11564391; doi:10.1007/s00405-024-08869-6)

Fig. S2

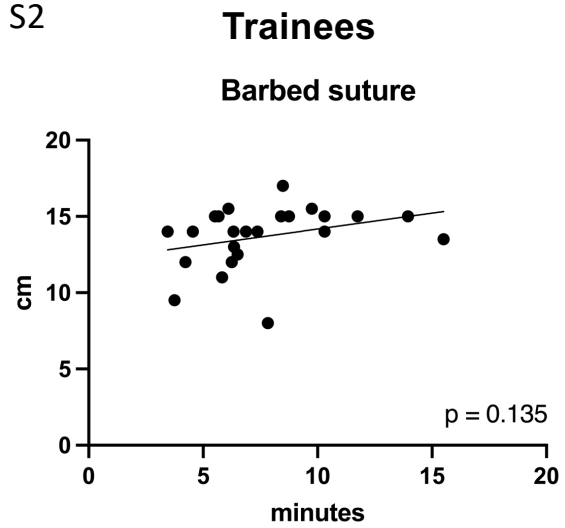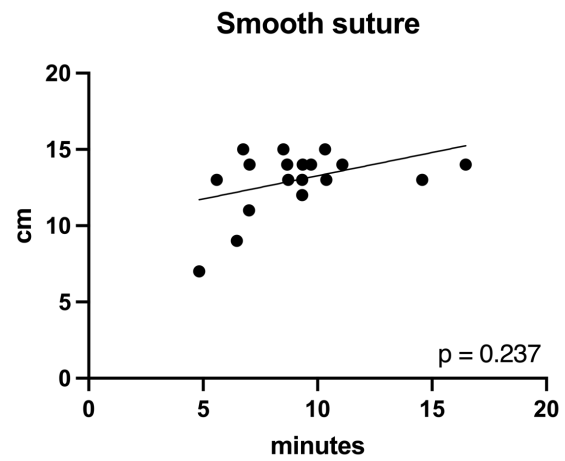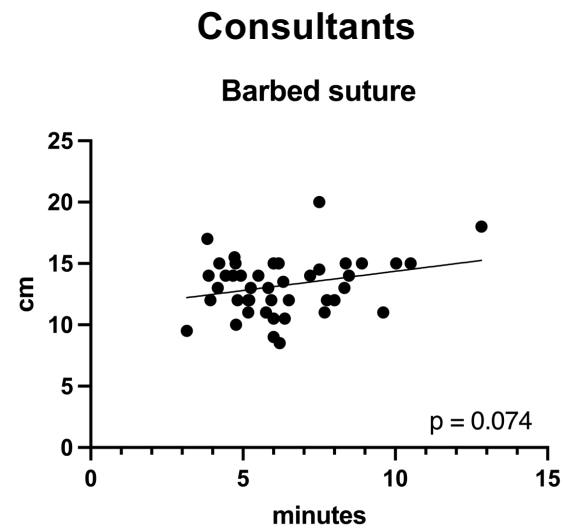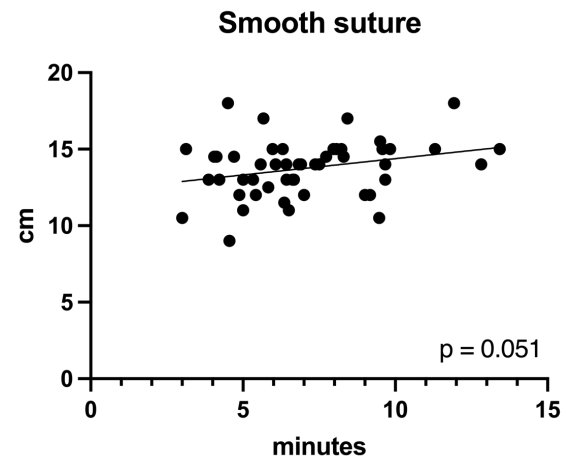

Supplement: Supplementary file 1 — Supplementary file1 (PDF 211 KB) [file 405_2024_8869_MOESM1_ESM.pdf]
